# Decontamination, pooling and dereplication of the 678 samples of the Marine Microbial Eukaryote Transcriptome Sequencing Project

**DOI:** 10.1186/s13104-021-05717-2

**Published:** 2021-08-09

**Authors:** Mick Van Vlierberghe, Arnaud Di Franco, Hervé Philippe, Denis Baurain

**Affiliations:** 1 InBioS – PhytoSYSTEMS, Eukaryotic Phylogenomics, University of Liège, Liège, Belgium; 2 Station D’Ecologie Théorique Et Expérimentale de Moulis, UMR CNRS 5321, Moulis, France

**Keywords:** Bioinformatics, Decontamination, Transcriptomes, Gene phylogenies, Phylogenomics, MMETSP, Algae, Eukaryotic evolution, Endosymbiotic gene transfer (EGT), Kleptoplastidy

## Abstract

**Objectives:**

Complex algae are photosynthetic organisms resulting from eukaryote-to-eukaryote endosymbiotic-like interactions. Yet the specific lineages and mechanisms are still under debate. That is why large scale phylogenomic studies are needed. Whereas available proteomes provide a limited diversity of complex algae, MMETSP (Marine Microbial Eukaryote Transcriptome Sequencing Project) transcriptomes represent a valuable resource for phylogenomic analyses, owing to their broad and rich taxonomic sampling, especially of photosynthetic species. Unfortunately, this sampling is unbalanced and sometimes highly redundant. Moreover, we observed contaminated sequences in some samples. In such a context, tree inference and readability are impaired. Consequently, the aim of the data processing reported here is to release a unique set of clean and non-redundant transcriptomes produced through an original protocol featuring decontamination, pooling and dereplication steps.

**Data description:**

We submitted 678 MMETSP re-assembly samples to our parallel consolidation pipeline. Hence, we combined 423 samples into 110 consolidated transcriptomes, after the systematic removal of the most contaminated samples (186). This approach resulted in a total of 224 high-quality transcriptomes, easy to use and suitable to compute less contaminated, less redundant and more balanced phylogenies.

## Objective

Plastid-bearing organisms are scattered across the eukaryotic tree. Among those, CASH lineages have plastids related to red algae, but the mechanisms by which they were acquired and the ancestors involved remain unclear [1–3]. To overcome the inconsistencies of purely endosymbiotic models [4], we propose kleptoplastidy [5] as an additional mechanism for explaining plastid spread among CASH lineages. In line with the shopping bag model [6], our hypothesis posits multiple transient interactions with preys of diverse origins but also proposes a rationale for the selective force driving the progressive accumulation of plastid-targeted genes. In such a scenario, also recently proposed by Bodyl [7], the phylogenetic diversity of plastid-targeted genes would be higher than predicted with endosymbiotic models, where genes originate mostly from a single source [8]. Obviously, testing this hypothesis requires a scrupulous phylogenetic study and at the largest scale possible. In addition, the inherent nature of those genes, which are transferred from one lineage to another, makes them hardly distinguishable from actual contaminations, prompting the use of data as clean as possible to avoid false positives. To overcome the limited diversity of complex algal proteomes and reduce the high redundancy of the richly sampled MMETSP transcriptomes [9, 10], we designed a consolidation pipeline. However, we chose to remove the most contaminated samples instead of trying to decontaminate them. This somewhat radical method has the advantage of allowing a more reliable interpretation of downstream phylogenetic analyses at the expense of a moderate loss of taxonomic breadth and richness. We used two methods to estimate contamination levels, one targeting cross-contaminations [11] (between MMETSP samples, using raw reads) and the other targeting more regular contaminations [12, 13] (from any eukaryotic source, using ribosomal markers). Finally, we decided to release the resulting transcriptomes in line with the FAIR data principles [14].

## Data description

MMETSP transcriptomic samples [9] cover a wide range of primary and complex algae and were recently re-assembled [10]. Despite showing better annotation overall, we observe high redundancy and contaminations, which complicates the study of plastid spread across eukaryotic lineages. To improve this dataset, we developed a pipeline to eliminate taxonomic redundancy while maximizing data purity and completeness. In detail, out of the 678 samples in MMETSP re-assemblies, 16 were discarded from the start (14 with less than 5000 sequences, 1 with a corrupted FASTQ file and another one because the organism was unknown). The remaining 662 were screened for contaminations with Forty-Two [12, 13] using 78 ribosomal protein markers, and 89 samples were further discarded because they were too contaminated (Data file 1, Data set 1), leaving 573 samples. To minimize taxonomic redundancy, we aimed to combine closely related samples. However, 124 samples represented unique genera and were not combined. As phylogenetic diversity can be very different across protist genera, we determined whether multiple samples within a given genus should be all combined or if some should only be combined at the species/strain level. To this end, we used CroCo [11] and combined the samples that shared a “cross-contamination” value ≥ 10% (interpreted here as close relatedness [11]) (Data file 2). Otherwise, they were left uncombined, even if from the same genus. This dual strategy yielded 110 combined samples (out of 423 candidate samples) and 26 uncombined samples (additional singletons), thereby aggregating 313 redundant samples (Data file 1, Data file 3). From a taxonomic point of view, this combination had the effect of reducing the number of samples by more than 50% for the majority of main phyla (Data file 4), resulting in a total of 260 transcriptomes (combined and singletons, Data set 2). Furthermore, the remaining most contaminated transcriptomes were removed using two strategies, (i) by targeting cross-contaminations with Sobek, a new parallel implementation of CroCo [11] (Data file 5), (ii) after removing intra-sample redundancy with CD-HIT-EST [15, 16] (sequence identity threshold of 95%), by targeting regular contaminations with Forty-Two (Data set 3) [12, 13]. Both detection methods combined pinpointed 36 transcriptomes, corresponding to 97 original samples (Data file 6, Data file 7, Data file 1). Finally, sample completeness was assessed with BUSCO [17, 18] (Data file 8). The resulting data set of 224 transcriptomes (Data set 2) spans a wide range of organisms, especially among photosynthetic species (172/224), with a majority of complex algae (145/224) (Data set 4). Its usefulness is illustrated with the GAPDH phylogeny (Data set 5). Briefly, two different trees were built after enriching the same starting set of sequences from high-quality proteomes [19], one using all 570 samples from plastid-bearing organisms and the other using 172 transcriptomes resulting from our pipeline. The comparison of these two trees shows that the enrichment performed with the data set described here is less contaminated, less redundant and more balanced from a taxonomic point of view, thereby allowing more robust evolutionary interpretations.

## Limitations

- The way some samples were combined at the genus level might lead to a loss of resolution at the species/strain level within a given genus.
- Even though combined samples were dereplicated, sequence redundancy remains substantial, especially among dinoflagellates, which are known for bearing multiple copies of their genes.
- Similarly, even if we provide a set of cleaned transcriptomes, contaminations remain. First, in some cases, such as ciliates, it is a “feature” of the phylum that all its members are highly contaminated. Therefore, we used a phylum-based outlier approach to determine whether a sample was too contaminated, because setting a global threshold for all phyla at once was impracticable. Second, in other cases, contamination by foreign sequences is a “necessary evil” for the reason that we plan to study transferred genes in a plastid acquisition context, and those would be undetectable in a completely “uncontaminated” transcriptome.

## Data Availability

The MMETSP re-assemblies analysed during this study are available in the Zenodo repository (https://zenodo.org/record/1212585/#.YA1WXdZ7lX0, https://doi.org/10.5281/zenodo.1212585, [9, 10]). All the remaining data generated or analysed during this study are publicly available in the figshare repository (https://doi.org/10.6084/m9.figshare.14079866.v5, https://doi.org/10.6084/m9.figshare.12362699.v1, https://doi.org/10.6084/m9.figshare.14727411.v3, https://doi.org/10.6084/m9.figshare.12154824.v3, https://doi.org/10.6084/m9.figshare.12213731.v3, https://doi.org/10.6084/m9.figshare.13634840.v1, https://doi.org/10.6084/m9.figshare.12410522.v3, https://doi.org/10.6084/m9.figshare.13006622.v1, https://doi.org/10.6084/m9.figshare.12173235.v3, https://doi.org/10.6084/m9.figshare.12998726.v3, https://doi.org/10.6084/m9.figshare.12154833.v3, https://doi.org/10.6084/m9.figshare.12401639.v1, https://doi.org/10.6084/m9.figshare.13096208.v2). Please see Table 1 and references [20–32] for details and links to the data.

**Table 1:** Overview of data files/data sets

Label	Name of data file/data set	File types (file extension)	Data repository and identifier (DOI or accession number)
Data file 1	Methods	PDF file (.pdf)	Figshare https://doi.org/10.6084/m9.figshare.14079866.v5 [20]
Data set 1	Forty-Two reports and configuration files (662 individual samples)	Text files (.tsv,.csv,.yaml)	Figshare https://doi.org/10.6084/m9.figshare.12362699.v1 [21]
Data file 2	Consolidation table	Spreadsheet (.xlsx)	Figshare https://doi.org/10.6084/m9.figshare.14727411.v3 [22]
Data file 3	Sample consolidation report	Image file (.pdf)	Figshare https://doi.org/10.6084/m9.figshare.12154824.v3 [23]
Data file 4	Redundancy drop analysis	Spreadsheet (.xlsx)	Figshare https://doi.org/10.6084/m9.figshare.12213731.v3 [24]
Data set 2	Transcriptomes	FASTA files (.tar.gz)	Figshare https://doi.org/10.6084/m9.figshare.13634840.v1 [25]
Data file 5	Sobek analysis summary	Text file (.csv)	Figshare https://doi.org/10.6084/m9.figshare.12410522.v3 [26]
Data set 3	Forty-Two reports and configuration files (260 transcriptomes)	Text files (.tsv,.csv,.yaml)	Figshare https://doi.org/10.6084/m9.figshare.13006622.v1 [27]
Data file 6	Consolidated sample purity (cross-contaminations)	Image file (.pdf)	Figshare https://doi.org/10.6084/m9.figshare.12173235.v3 [28]
Data file 7	Consolidated sample purity (contaminations)	Image file (.pdf)	Figshare https://doi.org/10.6084/m9.figshare.12998726.v3 [29]
Data file 8	Completeness analysis	Text file (.csv)	Figshare https://doi.org/10.6084/m9.figshare.12154833.v3 [30]
Data set 4	Taxonomic samplings	Image files (.png,.html,)	Figshare https://doi.org/10.6084/m9.figshare.12401639.v1 [31]
Data set 5	GAPDH phylogenies	Image files, text file (.pdf)	Figshare https://doi.org/10.6084/m9.figshare.13096208.v2 [32] Overview of data files/data sets

## Abbreviations

CASH: Cryptophytes, Alveolates, Stramenopiles, Haptophytes
MMETSP: Marine Microbial Eukaryote Transcriptome Sequencing Project
